# Increased levels of intramuscular cytokines in patients with jaw muscle pain

**DOI:** 10.1186/s10194-017-0737-y

**Published:** 2017-02-27

**Authors:** S. Louca Jounger, N. Christidis, P. Svensson, T. List, M. Ernberg

**Affiliations:** 1 Section for Orofacial Pain and Jaw Function, Department of Dental Medicine, Karolinska Institutet, SE 14104, Huddinge, Sweden; 2 Scandinavian Center for Orofacial Neurosciences (SCON), Huddinge, Sweden; 3 Section of Orofacial Pain and Jaw Function, School of Dentistry and Oral Health, Aarhus University, Vennelyst Boulevard 9, DK-8000 Aarhus C, Denmark; 4 Faculty of Odontology, Malmö University, Malmö, Sweden

**Keywords:** Cytokines, Bruxism, Masseter muscle, Myalgia, Temporomandibular disorders (TMD)

## Abstract

**Background:**

The aim of this study was to investigate cytokine levels in the masseter muscle, their response to experimental tooth-clenching and their relation to pain, fatigue and psychological distress in patients with temporomandibular disorders (TMD) myalgia.

**Methods:**

Forty women, 20 with TMD myalgia (Diagnostic Criteria for TMD) and 20 age-matched healthy controls participated. Intramuscular microdialysis was performed to sample masseter muscle cytokines. After 140 min (baseline), a 20-minute tooth-clenching task was performed (50% of maximal voluntary contraction force). Pain (Numeric rating scale 0–10) and fatigue (Borg’s Ratings of Perceived Exertion 6–20) were assessed throughout microdialysis, while pressure-pain thresholds (PPT) were assessed before and after microdialysis. Perceived stress (PSS-10) and Trait Anxiety (STAI) were assessed before microdialysis.

**Results:**

The levels of IL-6, IL-7, IL-8 and IL-13 were higher in patients than controls (Mann Whitney *U*-test; *P*’s < 0.05) during the entire microdialysis. IL-6, IL-8 and IL-13 changed during microdialysis in both groups (Friedman; *P*’s < 0.05), while IL-1β, IL-7 and GM-CSF changed only in patients (*P*’s < 0.01). IL-6 and IL-8 increased in response to tooth-clenching in both groups (Wilcoxon test; *P*’s < 0.05), while IL-7, IL-13 and TNF increased only in patients (*P*’s < 0.05). Patients had higher pain and fatigue than controls before and after tooth-clenching (*P* < 0.001), and lower PPTs before and after microdialysis (*P* < 0.05). There were no correlations between cytokine levels, pain or fatigue. Also, there were no differences in stress or anxiety levels between groups.

**Conclusions:**

In conclusion, the masseter levels of IL-6, IL-7, IL-8 and IL-13 were elevated in patients with TMD myalgia and increased in response to tooth-clenching. Tooth-clenching increased jaw muscle pain and fatigue, but without correlations to cytokine levels. This implies that subclinical muscle inflammation may be involved in TMD myalgia pathophysiology, but that there is no direct cause-relation between inflammation and pain.

## Background

Temporomandibular disorders (TMD) are the most common chronic pain conditions in the orofacial region, affecting approximately 10–15% of the adult population [1] and twice as many women as men [2]. The most common subtype is TMD myalgia with jaw muscle pain that is increased by function, pain on palpation, pain referral, restricted mouth opening, and headache [3, 4]. The etiology of TMD and the higher prevalence among women is not well understood.

One hypothesis is that excessive tooth-clenching/grinding might contribute by disturbing the local blood flow in overloaded muscles, leading to ischemia [5]. Epidemiological studies show greater odds of having TMD myalgia when self-reported tooth-clenching is present [6–8]. Ischemia releases neuroactive and inflammatory biomarkers, such as neuropeptides, bradykinin, protons, serotonin (5-HT), glutamate and cytokines that may activate and sensitize nociceptors on peripheral sensory afferents to induce muscle pain and allodynia [9, 10]. Repeated muscle activity may then maintain chronic muscle pain by temporal summation [11]. Previous studies have shown that intense chewing induced pain and fatigue in pain-free healthy participants with similar, but transient symptoms as in TMD myalgia patients [12–14]. This observation may suggest that, at least a subset of M-TMD pain patients could be more alike an exercise-induced muscle pain probably caused by ischemia and an accumulation of metabolic biomarkers in the masticatory muscles [12–14]. In another study, experimental tooth-clenching increased jaw muscle pain in patients with M-TMD and caused low levels of pain in controls. Patients with M-TMD had higher levels of 5-HT during the entire experiment, but did not increase in response to tooth-clenching, suggesting that other algesic substances might be released and activate nociceptors that are most likely already sensitized by 5-HT [15].

Studies have shown that several cytokines play a role in acute inflammatory muscle pain and in some chronic muscle pain conditions. There are both pro- and anti-inflammatory cytokines interacting with each other in a balanced matter in order to fight an infection and promote wound healing, and they are often released in a cascade in response to tissue damage. The pro-inflammatory cytokines can initiate an inflammatory response by recruiting other cytokines, macrophages, t-cells and b-cells, initiating the inflammatory response, while anti-inflammatory cytokines can reduce and promote healing by controlling the cytokine response [16].

For example, patients with various chronic pain conditions (neuropathic, nociceptive and mixed pain) had higher serum level of interleukin (IL)-6, IL-1β, IL-2, tumor necrosis factor (TNF), and interferon gamma (IFN-γ) than healthy controls, which correlated with pain intensity [17], and increased cerebrospinal fluid (CSF) and plasma level of IL-8 were reported in patients with widespread pain [18, 19]. In patients with TMD pain increased levels of IL-1β, IL-6, IL-10, TNF, IL-1ra, and monocyte attractant protein-1 (MCP-1) have been reported [19, 20].

Also, muscle levels of TNF and IL-1β were increased in trapezius trigger points in patients with myofascial pain [21, 22], and IL-6 increased in the painful trapezius muscle of patients with whiplash-associated disorders (WAD) [23]. Muscle and plasma levels of IL-6 and IL-8 increase in response to exercise [24, 25]. However, serum IL-10 showed a blunted response to exercise in fibromyalgia [26].

Regardless of their potential role in the development and maintenance of some chronic pain conditions, little is known about the peripheral involvement of cytokines in TMD myalgia. The aim of this study was therefore to compare the levels of the pro- and anti-inflammatory cytokines IL-1β, IL-2, IL-4, IL-5, IL-6, IL-7, IL-8, IL-10, IL-12, IL-13, TNF, IFN-γ, and GM-CSF in the masseter muscle between TMD myalgia patients and healthy controls and the effects of a repetitive tooth-clenching task on their release and relation to masseter pain intensity, fatigue, and pressure-pain thresholds (PPT). Furthermore, perceived stress and anxiety may also be involved in the pathophysiology of TMD pain [27]. Therefore, questionnaires were used to estimate the potential correlation between biomarkers and psychological variables.

We hypothesized that muscle level of the cytokines would be higher in patients than controls, increase in response to a repetitive tooth-clenching task in a similar manner in both groups, and correlate to pain intensity, fatigue and level of stress and anxiety.

## Methods

### Participants

Twenty women with TMD myalgia and twenty age-matched healthy women were included. Participants were recruited by advertisements and among colleagues and students at the Department of Dental Medicine at the Karolinska Institutet, Huddinge, Sweden where the study was performed. Inclusion criteria for the patients were age over 18, a diagnosis of myalgia according to the Diagnostic Criteria for TMD (DC/TMD) [1] and pain lasting for over 3 months. Inclusion criteria for the controls were age over 18, good general health and no history of/or current pain from the orofacial region. Exclusion criteria for both groups were systemic muscular or joint diseases, WAD, neuropathic pain or neurological disorders, pain of dental origin, and use of analgesics of non-steroidal anti-inflammatory drugs during 48 h before microdialysis.

Based on a previous study [15, 28] the power calculation showed that inclusion of 20 participants in each group would be sufficient to detect a significant difference of 20% (SD 30%) in biomarker levels with 80% power and a significance level of 5%.

The study followed the guidelines of the Declaration of Helsinki and was approved by the Regional Ethical Review Board in Stockholm (2009/2047-32), Sweden. All participants were given written and verbal information before participating and gave their written consent. The participants were compensated upon completion of their participation.

### Experimental protocol

**Fig. 1:**
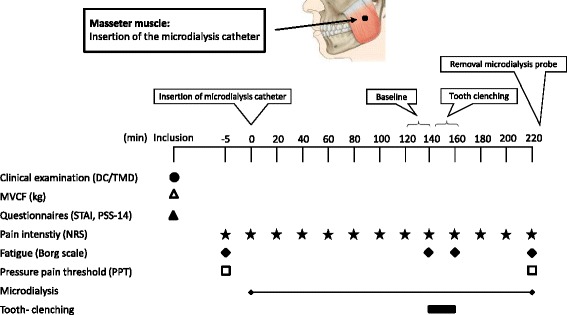
Flow chart of the experimental setting. The figure shows the time points (minutes) for the clinical examination (DC/TMD), assessment of MVCF (kg), questionnaires (STAI and PSS-14), pain intensity (NRS), fatigue (Borg scale), pressure pain threshold (PPT), microdialysis sampling and tooth-clenching in 20 women with temporomandibular disorder myalgia (Patients) and 20 pain-free healthy aged-matched women (Controls). DC/TMD = Diagnostic criteria for TMD; STAI = State-Trait Anxiety Inventory; PSS = Perceived Stress Scale

The study used a case–control design with one session lasting for 220 min. After inclusion, psychological distress was assessed; thereafter the maximal voluntary clenching force (MVCF), and PPT were recorded. Participants were reclined in a conventional dental chair and instructed to lie as still as possible during the experiment and to avoid talking. After baseline registrations and local anesthesia, microdialysis was performed in one of the masseter muscles. After 120-minutes (trauma phase), baseline assessments of pain intensity and fatigue were made (120–140 min), followed by a 20-minute clenching task (exercise). Pain intensity and fatigue were assessed after tooth-clenching. After an additional hour (recovery) the microdialysis catheter was removed and the PPTs were again assessed (Fig. 1).

### Experimental tooth-clenching

MVCF (kg) was assessed with a bite-force transducer (Aalborg University, Denmark) placed between the molars on the most suitable side, from a dental point of view. The same side was used for the clenching task and microdialysis. Participants were instructed to bite as hard as possible on the bite-force transducer for 2–3 s. The mean of three MVCF registrations was calculated. During the experimental tooth-clenching, participants were instructed to repeatedly bite 50% of their mean MVCF for 30-seconds followed by 30-seconds of rest during 20-minutes [15]. Visual feedback was used to maintain the clenching level.

### Assessments of pain intensity, fatigue and pressure-pain threshold

A 0–10 numerical rating scale (NRS) was used to assess pain intensity in the masseter muscle every 20^th^ minute during microdialysis. The Borg Rating of Perceived Exertion Scale (6–20; RPE) was used to measure fatigue [29].

PPT was assessed with an electronic pressure algometer (Somedic Sales AB, Höör, Sweden) with a standardized pressure rate of 50 kPa/s. The tip of the device was 1 cm^2^, covered with a 1-mm thick rubber-pad. PPT was recorded by applying pressure on the most prominent point of the masseter muscle during contraction, and on the right index finger, which was used as an extra-cranial reference point. Participants pressed a signal-button when the sensation of pressure turned into pain [30]. The mean of three registrations was used in statistical analyzes.

### Psychological distress

The Swedish version of the State-Trait Anxiety Inventory (STAI) was used to assess trait-anxiety. It contains twenty questions measuring the levels of anxiety as a personal characteristic. Scores ranges from 20 to 80, where high scores (>30) indicate higher levels of anxiety [31, 32].

Stress levels were measured with the Swedish version of the Perceived Stress Scale-14 (PSS-14) consisting of 14 stress-related questions of a general nature, including feelings and thoughts during the last month, situations in life that are perceived as stressful, and the current level of experienced stress. The total scoring is 56, and scores below 23.67 are considered normal in healthy participants [33, 34].

### Microdialysis

The most prominent point of the masseter muscle, verified by palpation during contraction, was chosen for microdialysis. The skin overlying the muscle was anaesthetized with a local injection (0.5 ml) of Lidocaine (Xylocaine 20 mg /ml), carefully avoiding anaesthetizing the underlying muscle.

A sterile split able introducer (CMA Microdialysis AB, Solna, Sweden) was inserted intramuscularly in parallel to the muscle fibers at a 45-degree angle to a depth of approximately 40 mm from the skin surface [15, 35]. After approximately 20 mm, a slight resistance occurred, typically felt upon penetration of the muscle fascia, and the introducer was additionally inserted 20 mm. A sterile 100 kDa 60 mm long microdialysis catheter with 20 mm membrane length (CMA71 Microdialysis AB, Solna, Sweden) was inserted to the full length, thereafter the introducer was removed by splitting the plastic tube. The catheter was perfused (5 μL/ min) with a Ringer-Acetate solution (Pharmacia & Upjohn, Copenhagen, Denmark) containing 3 mmol glucose and 0.5 mmol lactate from a 2-ml syringe connected to a micro-infusion pump (CMA107, Microdialysis AB, Solna, Sweden). Samples (100 μL) were collected every 20-minutes in capped microvials and immediately frozen (−80 °C). After microdialysis, the catheter was removed and the membrane was checked to ensure that no damaged had occurred.

### Analyzes of dialysate

The cytokines were analyzed with Luminex technology using multiplex immunoassay panels (Milliplex®map kit, Human High sensitivity, T-cell magnetic bead panel, 96-well plate assay, Merck Millipore Darmstadt, Germany) in accordance to the manufacturer’s manual.

The limit of detection (LOD) for each cytokine respectively were for IL-1β, IL-2, IL-5, and IL-12: 0.49 pg./mL; IL-4: 1.83 pg./mL; IL-6: 0.18 pg./mL; IL-7: 0.37 pg./mL; IL-8: 0.31 pg./mL, IL-10: 1.46 pg./mL; IL-13: 0.24 pg./mL; GM-CSF: 1.22 pg./mL; IFN-γ: 0.61 pg./mL and for TNF: 0.43 pg./mL.

For each cytokine the number of samples with levels below LOD were calculated. As a quality control, cytokines with >50% of samples below LOD were excluded from further analysis. This was based on findings from our group that some cytokines are undetectable in many individuals, making the results difficult to interpret (Ernberg et al, personal communication). Others have used similar approaches, although with less strict criteria [36].

### Statistics

Data were analyzed with SigmaPlot for Windows, version 11 (Systat Software Inc. Chicago, IL, USA) and STATISTICA, StatSoft Dell Software version 12.0 (Round Rock, Texas USA). For descriptive statistics mean and standard deviation (SD) or median and interquartile range (IQR) were used. Non-parametric statistics were used since most cytokines were not normally distributed and attempts to transform data did not change this. To analyze differences in cytokine levels between groups, the average of all 11 dialysate samples (0–220 min) was calculated and compared with Mann-Whitney *U*-test. Friedman-test was used to analyze changes in cytokine levels over time. Wilcoxon-test compared the time points 140 min (BL) and 160–220 min within groups. Cytokine, pain and fatigue levels after clenching (160 min) were compared between groups with Mann-Whitney *U*-test. Spearman correlations-test with Bonferroni correction for multiple testing was used to analyze correlations between cytokine levels, pain, fatigue and psychological distress. Differences in STAI-trait and PSS-14 scores between groups and differences in PPTs between sides before and after microdialysis were analyzed with unpaired *t*-test. The level of significance was *P* < 0.05.

## Results

### Cytokine levels

IL-2, IL-4, IL-5, IL-10 and IFN-γ were excluded from further statistical analysis since more than 50% of the samples were below LOD (Table 1).

**Table 1:** Dialysate samples with cytokines levels below LOD

	IL-1β	IL-2	IL-4	IL-5	IL-6	IL-7	IL-8	IL-10	IL-12	IL-13	TNF	IFN-γ	GM-CSF
All	75 (19.1)	268 **(68.4)**	291 **(74.2)**	264 **(67.3)**	68 (17.3)	133 (33.9)	45 (11.5)	210 **(53.6)**	180 (45.9)	140 (35.7)	169 (43.1)	319 **(81.4)**	178 (45.4)
Patients	54 (24.3)	133 (59.9)	188 (84.7)	144 (64.9)	38 (17.1)	82 (36.9)	18 (8.1)	118 (53.1)	127 (57.2)	64 (28.8)	100 (45.0)	165 (74.3)	118 (53.2)
Controls	21 (12.4)	135 (79.4)	103 (60.6)	120 (70.6)	30 (17.6)	51 (30.0)	27 (15.9)	92 (53.6)	53 (31.2)	76 (44.7)	69 (40.6)	154 (90.6)	60 (35.3)

Table show the dialysate samples with cytokines levels below LOD in 20 women with Temporomandibular Disorders myalgia (Patients) and 20 pain-free healthy aged-matched women (Controls). Data are presented as n = number of undetectable dialysate samples (%)

*LOD* Limits of detection, *IL* Interleukin, *TNF* Tumor necrosis factor, *IFN* Interferon, *GM-CSF* Granulocyte macrophage colony-stimulating factor. Bold figures denote cytokines with 50% of the samples below LOD (excluded from further analyses)

**Fig. 2:**
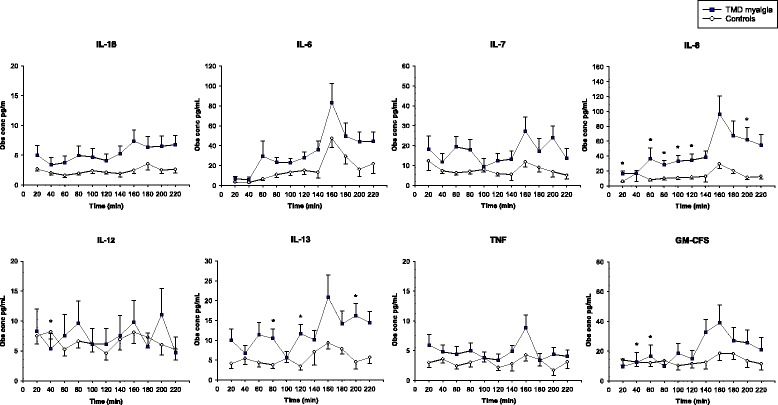
The levels of cytokines during microdialysis. Graph showing the levels of cytokines (mean and SEM) during microdialysis in 20 women with temporomandibular disorder myalgia (Patients) and 20 pain-free healthy aged-matched women (Controls). After 140 min (baseline), a 20-minute repetitive tooth-clenching task (140–160 min) was performed followed by one-hour rest (160–220 min). IL-1β (*P* = 0.0007), IL-6 (*P < 0.001*), IL-7 (*P* = 0.012), IL-8 (*P < 0.001*), IL-13 (*P* < 0.001), and GM-CSF (*P* = 0.008) increased with time in the patients, whereas IL-6 (*P* < 0.001), IL-8 (*P < 0.001*), and IL-13 (*P* = 0.042) increased with time in the controls (Friedman test). IL-6 (*P* < 0.001), IL-8 (*P* = 0.001), IL-13 (*P* = 0.035), and TNF (*P* = 0.042) had increased after clenching (160 min) compared to baseline (140 min) in the patients, and IL-6 (*P* = 0.004) and IL-8 (*P* = 0.032) had increased in the controls (Wilcoxon test). There were no significant differences in cytokine levels between patients and controls after clenching (160 min), although there was a trend for IL-8 (Mann-Whitney *U*-test, *P* = 0.074). *= significant difference between the groups (*P* < 0.05)

The dialysate levels of the cytokines are shown in Table 2 and Fig. 2. The dialysate levels of IL-6, IL-7, IL-8, and IL-13 were higher in patients than controls during the entire microdialysis. IL-6, IL-8 and IL-13 changed during microdialysis in both groups, while IL-1β, IL-7 and GM-CSF changed only in patients. IL-6 and IL-8 increased in response to tooth-clenching in both groups, while IL-7, IL-13 and TNF only increased in the patients. There were no significant differences in cytokine levels between patients and controls after tooth-clenching (160 min).

**Table 2:** The average cytokine levels for all samples combined

	IL-1β	IL-6	IL-7	IL-8	TNF	GM-CSF	IL-12	IL-13
Dialysate levels								
Patients	2.3 (7.0)	25.8 (42.3)	11.4 (21.5)	36.2 (51.4)	4.4 (8.8)	10.1 (11.2)	5.5 (11.8)	4.1 (5.9)
Controls	1.6 (2.3)	11.0 (12.6)	6.4 (3.2)	10.1 (14.7)	3.5 (1.5)	14.8 (23.3)	7.4 (3.0)	9.7 (13.4)
*P-values*	*0.208*	***0.015***	***0.041***	***0.002***	*0.291*	*0.199*	*0.507*	***0.002***

The average cytokine levels for all samples combined (0–220 min) in 20 women with Temporomandibular Disorders myalgia (Patients) and 20 pain-free healthy age-matched women (Controls). Data are presented as median (IQR)

*IQR* interquartile range (75 percentile minus 25 percentile), *IL* Interleukin, *TNF* Tumor necrosis factor, *GM-CSF* Granulocyte macrophage colony-stimulating factor. Bold italic figures denote significant group differences (*P* < 0.05)

### Pain intensity and levels of fatigue

Patients had higher baseline pain intensity and fatigue than controls. Tooth-clenching increased pain intensity in patients and evoked mild pain in controls. It also increased fatigue in both groups (Table 3).

**Table 3:** The median (IQR) pain intensity (NRS) and fatigue (Borg RPE) in the masseter muscle

	Before	After	*P-*values
Pain			
Patients	3 (3)	7 (3)	***<0.001***
Controls	0 (0)	0 (2)	***0.016***
*P-values between groups*	***<0.001***	***<0.001***	
Fatigue			
Patients	13 (4)	19 (1)	***<0.001***
Controls	6 (0)	14 (3)	***<0.001***
*P-values between groups*	***<0.001***	***<0.001***	

Table show the median pain intensity and fatigue before and after a 20-minute tooth-clenching task in 20 women with Temporomandibular Disorders myalgia (Patients) and 20 pain-free healthy age-matched women (Controls). *IQR* interquartile range (75 percentile minus 25 percentile), *NRS* Numeric rating scale (0–10), *Borgs’s RPE* Borg’s ratings of perceived exertion scale (6–20). Bold italic figures denote significant differences (*P* < 0.05)

There were no significant correlations between cytokines levels and other variables (*P* > 0.102; r_s_ < 0.384).

### Baseline characteristics and PPT

Baseline characteristics of the participants and PPT before and after microdialysis are shown in Table 4. There were no differences between groups in PSS-14 or STAI-trait levels, but the MVCF was lower in patients than controls. PPT over the masseter muscles were lower in patients than controls, in contrast to the reference point, but had not changed after microdialysis in any group (Table 4).

**Table 4:** Baseline characteristics in 20 women with Temporomandibular Disorders myalgia and 20 healthy pain-free age-matched women

	Patients	Controls	*P-*values
Age (yr)	31 (10)	29 (11)	
MVCF (kg)	356 (205)	469 (113)	***0.018***
STAI-trait (20–80)	42 (19)	36 (10)	0.321
PSS-14 (0–56)	23 (15)	20 (9)	0.392
PPT (kPa)			
Before			
Experimental side	160 (±70)	239 (±62)	***0.003***
Control side	163 (±46)	230 (±30)	***<0.001***
*P-values between sides*	*0.876*	*0.570*	
Reference point	412 (±131)	384 (±115)	0.573
After			
Experimental side	124 (±71)	232 (±52)	***<0.001***
Control side	147 (±66)	234 (±48)	***<0.001***
*P-values between sides*	*0.298*	*0.919*	
Reference point	454 (±273)	425 (±94)	0.741

The baseline characteristics as well as pressure pain thresholds (PPT) before and after microdialysis are shown

Data are presented as median (IQR) or mean (±SD). *IQR* interquartile range (75 percentile minus 25 percentile), *SD* Standard deviation, *MVCF* Maximum voluntary contraction force, *STAI* State- and trait anxiety inventory, *PSS* Perceived stress scale. Bold italic figures denote significant differences (*P* < 0.05)

## Discussion

The main findings were that the masseter levels of IL-6, IL-7, IL-8 and IL-13 were higher in TMD myalgia patients than controls and that repetitive tooth-clenching increased the levels of IL-6 and IL-8 in both groups, while IL-7, IL-13 and TNF increased only in patients. Also, tooth-clenching evoked higher pain intensity and fatigue in TMD myalgia patients than controls. Finally, there were no correlations between pain, fatigue and cytokine levels.

Overall, the results from this study supports the hypotheses that 1) the muscle levels of pro- and anti-inflammatory cytokines appear to be elevated in TMD myalgia and 2) severally pro- and anti-inflammatory cytokines increased after repetitive tooth-clenching in both groups. However, there were no significant differences between TMD myalgia patients and controls. This lends support for a role of peripheral inflammation to drive chronic muscle pain. However, 3) no correlations with pain, fatigue or PPT levels were found, so in future studies the balance and interaction between pro- and anti-inflammatory cytokines as well as other inflammatory mediators need to be considered.

This is the first study to show elevated muscle levels of the pro-inflammatory cytokines IL-6 and IL-8 and the anti-inflammatory cytokines IL-7 and IL-13 in painful jaw muscles of patients with TMD myalgia which supports the hypothesis that patients with TMD myalgia have higher levels of cytokines. The results are partly consistent with previous results in other localized myalgias showing increased IL-6 in the painful trapezius muscle of WAD patients [23] and IL-1β and TNF in myofascial trapezius trigger points [21, 22], although one early study did not find any difference in trapezius levels of IL-6 between patients with chronic trapezius myalgia and controls [37]. They are also consistent with very recent results showing that lipopolysaccharide (LPS) stimulated monocytes from patients with TMD show an enhanced production of IL-1β, TNF and IL-6 [38]. Pro-inflammatory cytokines may act directly on nociceptor terminals to induce pain [39]. Increased muscle levels of other inflammatory markers, such as serotonin and glutamate have also been reported in chronic localized myalgia [15, 23, 35]. These results combined gives support for the involvement of peripheral mechanisms in localized chronic myalgia, and may implicate that patients with chronic pain constantly have their immune system switched on with higher levels of inflammatory mediators leading to peripheral sensitization, which may drive central sensitization processes and pain [40]. The increased muscle levels of IL-7 and IL-13 in the patients in this study imply that anti-inflammatory cytokines are locally produced to counteract this effect, although this may not be sufficient as LPS-stimulated monocytes from TMD-patients showed a blunted response of IL-10 [38].

In patients with TMD elevated plasma levels of both pro- (IL-1β, IL-6, TNF) and anti-inflammatory (IL-10) cytokines were recently reported [20]. Contrary, in TMD patients with widespread pain and in fibromyalgia plasma levels of pro-inflammatory IL-8 were higher [18, 19, 41] and IL-10 lower than controls [18]. This suggests that the balance between pro- and anti-inflammatory cytokines may differ between TMD with and without widespread pain [19] and supports a shift to a more pronounced central pro-inflammatory state in widespread myalgia compared to localized myalgia. Thus, peripheral sensitization may be of greater importance in the pathophysiology of localized myalgias than in widespread pain.

Tooth-clenching increased IL-6, IL-7, IL-8, TNF and IL-13 in TMD myalgia patients and IL-6 and IL-8 in controls, in line with previous studies [25, 37]. IL-6 and IL-8 are regarded as myokines, i.e. cytokines that are produced in the muscle [24], while TNF probably is produced in other tissue [42]. Although plasma cytokines were not analyzed in this study, muscle levels of IL-6 and IL-8 (but not IL-1β or TNF) were reported higher than plasma levels in patients with fibromyalgia, supporting that they are produced in the muscle [25]. It is well described that plasma levels of IL-6 increase in response to exercise. Also, plasma levels of the anti-inflammatory cytokines IL-4, IL-7, IL-10, and IL-13 seem to increase after exercise in healthy subjects [43] and IL-10 also in patients with knee-osteoarthritis [44]. On the contrary, in patients with fibromyalgia, plasma IL-10 showed a blunted response and IL-8 seem to decrease [26, 45]. Thus, patients with localized pain may be able to recruit an anti-inflammatory response after exercise in contrast to patients with generalized pain.

The repetitive tooth-clenching task evoked pain in the controls and increased fatigue in both groups, with higher intensities in TMD myalgia patients in accordance to previous studies using the same methodology [15, 30]. However, other mechanisms than release of cytokines are most probably responsible for this as there were no correlations between the release of cytokines on one hand, and pain or fatigue on the other. This indicates that there is no direct cause-effect relation between pain and cytokine levels. Indeed, the increased pain level may be an effect of the release of other biomarkers [35, 46] or interactions between several other mechanisms [47].

Pain on palpation, reflecting muscle allodynia, is one of the key symptoms of chronic muscle pain why it was not surprising that PPT were lower in patients than controls. Allodynia is generally regarded a sign of central sensitization [48], but peripheral sensitization may participate to the lowered PPT, as there were no differences between TMD myalgia patients and controls in PPT over the reference point. However, masseter PPT did not change in response to tooth-clenching and did not correlate with cytokine levels. This could be the reason why the influence of muscle cytokines on muscle allodynia in TMD myalgia is probably minor, if any at all.

### Limitations and strengths

This is, to our knowledge, the first study investigating the interstitial jaw-muscle release of cytokines in TMD myalgia. Another strength is that a panel with 13 pro- and anti-inflammatory cytokines was used for the analysis in the same dialysate samples so that differences in their pattern after tooth-clenching could be analyzed.

One limitation was that some cytokines were under LOD and had to be excluded. Yet, the most common cytokines reported in previous microdialysis studies could be detected. There are several factors that can affect the dialysate levels, such as the flow-rate, the diffusion-rate through the tissue, the area and weight cut-off of the dialysis membrane, and the composition of the perfusate [49]. Perhaps by adding a colloid to the ringer-solution, the cytokines under LOD could have been detected [50]. Another limitation was that the relative recovery was not analyzed, which could have provided a true value of the extracellular concentrations. Furthermore, the patients with TMD myalgia were quite young and had a low average pain intensity at baseline, and may therefore not fully represent the general TMD myalgia population. However, the variability in pain intensity and age differs in experimental studies of TMD patients with myalgia. The data in our study is in accordance with findings of a previous study [51]. Finally, the sample was limited only to women that were psychologically healthy why it is unclear if the results can be generalized to male patients and patients with more severe psychological co-morbidities.

## Conclusion

This study showed that the muscle levels of IL-6, IL-7, IL-8 and IL-13 were increased in patients with TMD myalgia and increased further in response to experimental tooth-clenching, as did jaw-muscle pain and fatigue. This give further support that muscle inflammation may drive chronic myalgia, but that patients with TMD myalgia have a normal anti-inflammatory response to exercise. However, the lack of correlation between pain and cytokine levels, indicates that there is no direct cause-relation effect between increased pain and cytokine release and that other peripheral mediators and mechanisms, such as central sensitization are important for pain mediation.

## Abbreviations

5-HT: 5-hydroxytryptamine; serotonin
DC/TMD: Diagnostic Criteria for temporomandibular disorders
IFN-γ: Interferon gamma
IL: Interleukin
NRS: Numeric rating scale
PPT: Pressure pain threshold
PSS: Perceived Stress Scale
STAI-trait: State-Trait Anxiety Inventory
TMD: Temporomandibular disorders
TNF: Tumor necrosis factor
WAD: Whiplash-associated disorders
